# Supplementary material for: Quality of antenatal care and its potential impacts on delivery services and postnatal care compliance among reproductive women in Bangladesh: A situation analysis from the Bangladesh Demographic and Health Survey 2017
Source: PLoS One. 2025 Apr 29;20(4):e0321945. doi: 10.1371/journal.pone.0321945 (PMC12040109; doi:10.1371/journal.pone.0321945)
Supplement: S1 Table — (DOCX) [file pone.0321945.s001.docx]

**S1 Table: Association between outcomes and exposures, unadjusted risk ratio (95% CI)**

| **Background characteristics** | **Facility Delivery** | **Delivery by SBA** | **PNC for mother within 48 hours** | **PNC for child within 48 hours** |
| --- | --- | --- | --- | --- |
| **Quality of ANC** |  |  |  |  |
| No | Ref | Ref | Ref | Ref |
| Yes | 1.8 (1.69 - 1.87) | 1.7(1.65 - 1.8) | 1.7 (1.63 - 1.78) | 1.7 (1.63 - 1.78) |
| **Mother's age** |  |  |  |  |
| <19y | Ref | Ref | Ref | Ref |
| 20-29y | 1(0.92 - 1.07) | 1(0.91 - 1.05) | 1(0.9 - 1.02) | 1(0.91 - 1.04) |
| 30-39y | 0.9(0.84 - 1.01) | 0.9(0.83 - 0.99) | 0.9(0.83 - 0.99) | 0.9(0.83 - 0.99) |
| 40-49y | 0.9(0.69 - 1.23) | 1(0.75 - 1.25) | 0.9(0.74 - 1.23) | 1(0.74 - 1.24) |
| **Mother's education** |  |  |  |  |
| No education (0 years) | Ref | Ref | Ref | Ref |
| Primary education (1-5 years) | 1.2(1.03 - 1.47) | 1.2(1 - 1.4) | 1.2(0.98 - 1.37) | 1.2(1.01 - 1.4) |
| Secondary incomplete (6-9 years) | 1.9(1.62 - 2.28) | 1.9(1.62 - 2.22) | 1.9(1.59 - 2.17) | 1.9(1.59 - 2.18) |
| Secondary complete or higher (10+ years) | 2.9(2.43 - 3.39) | 2.7(2.33 - 3.18) | 2.6(2.24 - 3.04) | 2.6(2.26 - 3.08) |
| **Mother’s occupation** |  |  |  |  |
| Not working | Ref | Ref | Ref | Ref |
| Agricultural | 0.7(0.62 - 0.72) | 0.7(0.65 - 0.75) | 0.7(0.65 - 0.75) | 0.7(0.65 - 0.75) |
| Skilled/unskilled manual | 1(0.9 - 1.09) | 1(0.92 - 1.1) | 1(0.92 - 1.1) | 1(0.93 - 1.1) |
| Service/sales | 0.8(0.67 - 0.95) | 0.8(0.68 - 0.94) | 0.8(0.72 - 0.98) | 0.8(0.72 - 0.98) |
| **Husband’s occupation** |  |  |  |  |
| Not working | Ref | Ref | Ref | Ref |
| Agricultural | 0.7(0.54 - 0.78) | 0.7(0.56 - 0.79) | 0.7(0.56 - 0.78) | 0.7(0.57 - 0.8) |
| Skilled/unskilled manual | 1.3(1.15 - 1.4) | 1.3(1.16 - 1.4) | 1.3(1.14 - 1.37) | 1.3(1.16 - 1.39) |
| Service/sales | 1.4(1.27 - 1.56) | 1.4(1.28 - 1.56) | 1.4(1.25 - 1.52) | 1.4(1.26 - 1.54) |
| **Birth order** |  |  |  |  |
| 1 | Ref | Ref | Ref | Ref |
| 2-3 | 0.8(0.73 - 0.82) | 0.8(0.73 - 0.81) | 0.8(0.73 - 0.81) | 0.8(0.74 - 0.82) |
| 4+ | 0.4(0.39 - 0.51) | 0.5(0.41 - 0.52) | 0.5(0.43 - 0.54) | 0.5(0.42 - 0.54) |
| **Residence** |  |  |  |  |
| Urban | Ref | Ref | Ref | Ref |
| Rural | 0.7(0.67 - 0.75) | 0.7(0.67 - 0.74) | 0.7(0.68 - 0.75) | 0.7(0.68 - 0.75) |
| **Division** |  |  |  |  |
| Barishal | Ref | Ref | Ref | Ref |
| Chittagong | 1.2(1.02 - 1.4) | 1.1(0.94 - 1.23) | 1.1(0.96 - 1.26) | 1.1(0.97 - 1.27) |
| Dhaka | 1.5(1.26 - 1.71) | 1.3(1.12 - 1.45) | 1.3(1.12 - 1.46) | 1.3(1.13 - 1.47) |
| Khulna | 1.6(1.33 - 1.84) | 1.3(1.17 - 1.55) | 1.4(1.2 - 1.58) | 1.4(1.2 - 1.59) |
| Mymensingh | 1(0.82 - 1.2) | 0.9(0.74 - 1.03) | 0.9(0.75 - 1.04) | 0.9(0.75 - 1.05) |
| Rajshahi | 1.3(1.14 - 1.59) | 1.2(1 - 1.33) | 1.2(1.02 - 1.35) | 1.2(1.03 - 1.37) |
| Rangpur | 1.2(1.03 - 1.44) | 1(0.89 - 1.21) | 1(0.9 - 1.21) | 1.1(0.9 - 1.22) |
| Sylhet | 1(0.83 - 1.22) | 0.9(0.72 - 1.01) | 0.9(0.73 - 1.03) | 0.9(0.75 - 1.05) |
| **Wealth quintile** |  |  |  |  |
| Poor | Ref | Ref | Ref | Ref |
| Poorest | 1.4(1.25 - 1.61) | 1.5(1.28 - 1.64) | 1.4(1.26 - 1.61) | 1.5(1.3 - 1.65) |
| Middle | 1.9(1.64 - 2.09) | 1.9(1.69 - 2.13) | 1.9(1.69 - 2.11) | 1.9(1.71 - 2.14) |
| Rich | 2.3(2.03 - 2.55) | 2.3(2.03 - 2.53) | 2.2(1.99 - 2.46) | 2.2(2 - 2.48) |
| Richest | 3(2.68 - 3.32) | 3(2.68 - 3.29) | 2.9(2.62 - 3.2) | 2.9(2.63 - 3.22) |
